# Supplementary material for: Protective Effect of Dexmedetomidine against Hyperoxia-Damaged Cerebellar Neurodevelopment in the Juvenile Rat
Source: Antioxidants (Basel). 2023 Apr 21;12(4):980. doi: 10.3390/antiox12040980 (PMC10136028; doi:10.3390/antiox12040980)
Supplement: Supplementary file 1 [file antioxidants-12-00980-s001.zip › Table S1. Quantification of Purkinje cells molecular layer.pdf]

Table S1. Quantification of co-labeled Calbindin (Calb1) and DAPI positive Purkinje cells (PC) of the cerebellar molecular layer and molecular layer (ML) depth

	NO	NOD	HY	HYD
**hyperoxia**	–	–	+	+
**dexmedetomidine**	–	+	–	+
**P7**				
**Calbindin+ (% of control)**	100±8.3	96±7.5	72±5.8	101±6.9
**Calbindin+ (cell count/ROI)**	9.5±0.79	9.1±0.71	6.8±0.54	9.6±0.66
**Molecular layer depth**	100±3.0	85±3.3	75±2.3	90±2.9
**P9**				
**Calbindin+ (% of control)**	100±6.8	121±9.0	74±4.5	119±5.2
**Calbindin+ (cell count/ROI)**	6.0±0.41	7.3±0.54	4.4±0.27	7.1±0.31
**Molecular layer depth**	100±4.2	107±3.3	93±5.1	103±0.7
**P11**				
**Calbindin+ (% of control)**	100±5.0	98±3.3	84±4.4	112±8.2
**Calbindin+ (cell count/ROI)**	5.8±0.29	5.6±0.19	4.8±0.26	6.5±0.47
**Molecular layer depth**	100±3.8	102±2.8	81±2.2	105±4.6
**P14**				
**Calbindin+ (% of control)**	100±4.8	80±4.3	91±7.0	73±8.3
**Calbindin+ (cell count/ROI)**	5.1±0.25	4.1±0.22	4.7±0.36	3.8±0.42
**Molecular layer depth**	100±5.1	95±3.2	90±6.9	108±4.2

Data are normalized to the level of rat pups exposed to normoxia at each time point (control 100 %) and the 100 % values are 9.5 (P7), 6.0 (P9), 5.8 (P11), and 5.1 (P14) cells per regions of lobules or 1.5 (P7), 1.4 (P9), 3.1 (P11), and 3.6 (P14) length of molecular layer, respectively. n = 6/group. *$p < 0.05$, **$p < 0.01$, ***$p < 0.001$, ****$p < 0.0001$ (ANOVA, Bonferroni's *post hoc* test).
