# Supplementary material for: Protective Effect of Dexmedetomidine against Hyperoxia-Damaged Cerebellar Neurodevelopment in the Juvenile Rat
Source: Antioxidants (Basel). 2023 Apr 21;12(4):980. doi: 10.3390/antiox12040980 (PMC10136028; doi:10.3390/antiox12040980)
Supplement: Supplementary file 1 [file antioxidants-12-00980-s001.zip › Table S2. Quantitation of Pax6 and PCNA.pdf]

Table S2. Quantitation of Pax6 and PCNA single and double positive cells of the cerebellar molecular layer.

	NO	NOD	HY	HYD
**hyperoxia**	–	–	+	+
**dexmedetomidine**	–	+	–	+
**P7**				
**Pax6+**	100±8.1	97±7.7	64±3.3	94±2.9
**PCNA+**	100±9.4	79±6.4	57±2.4	81±1.7
**Pax6/PCNA+**	100±6.8	93±4.8	52±2.5	90±2.7
**P9**				
**Pax6+**	100±5.7	102±11.3	93±5.8	100±11.0
**PCNA+**	100±8.5	86±6.0	91±3.2	95±5.8
**Pax6/PCNA+**	100±7.4	62±6.3	69±5.2	100±8.2
**P11**				
**Pax6+**	100±4.0	88±6.3	87±4.1	99±6.8
**PCNA+**	100±7.0	97±6.0	74±6.2	88±7.1
**Pax6/PCNA+**	100±4.0	99±5.9	52±2.8	101±5.0
**P14**				
**Pax6+**	100±7.3	116±6.6	85±7.0	93±7.3
**PCNA+**	100±6.0	94±7.9	79±3.6	108±10.3
**Pax6/PCNA+**	100±5.3	61±4.4	49±3.2	102±6.2

Data are normalized to the level of rat pups exposed to normoxia at each time point (control 100 %) and the 100 % values are 61.1 (P7), 67.6 (P9), 63.0 (P11), and 67.2 (P14) Pax6+ cells, and 41.9 (P7), 47.8 (P9), 47.8 (P11), and 47.9 (P14) PCNA+ cells, or 34.1 (P7), 49.8 (P9), 12.3 (P11), and 13.6 (P14) Pax6+/PCNA+ cells per regions of lobules, respectively. n = 6/group. *$p < 0.05$, **$p < 0.01$, ***$p < 0.001$, ****$p < 0.0001$ (ANOVA, Bonferroni's *post hoc* test; Kruskal-Wallis, Dunn´s *post hoc* test).
