# Supplementary material for: Protective Effect of Dexmedetomidine against Hyperoxia-Damaged Cerebellar Neurodevelopment in the Juvenile Rat
Source: Antioxidants (Basel). 2023 Apr 21;12(4):980. doi: 10.3390/antiox12040980 (PMC10136028; doi:10.3390/antiox12040980)
Supplement: Supplementary file 1 [file antioxidants-12-00980-s001.zip › Table S3. Quantification of cerebellar homogenates for Purkinje cells factors and neurotrophins.pdf]

Table S3. Quantification of cerebellar homogenates for Purkinje cells−associated mediators and neurotrophins.

	NO	NOD	HY	HYD
**hyperoxia**	−	−	+	+
**dexmedetomidine**	−	+	−	+
**P7**				
Calb1	100±6.9	60±8.2	68±8.2	154±10.1
Shh	100±8.6	41±7.9	48±7.9	46±2.8
BDNF	100±11.8	96±12.8	57±5.5	56±8.5
NGF	100±7.4	88±7.0	73±2.0	121±12.3
NT3	100±4.8	81±4.7	56±10.2	52±10.0
**P9**				
Calb1	100±6.4	62±5.3	64±7.2	87±9.8
Shh	100±7.4	122±4.8	69±6.1	81±5.4
BDNF	100±12.7	109±6.8	102±5.3	126±7.2
NGF	100±8.1	98±12.6	76±7.2	90±8.0
NT3	100±3.3	71±7.0	121±7.6	95±9.0
**P11**				
Calb1	100±4.9	162±11.2	204±13.7	148±12.6
Shh	100±5.4	90±4.5	76±4.4	99±7.6
BDNF	100±9.2	81±7.3	90±10.1	110±12.9
NGF	100±6.6	77±9.2	121±10.0	152±21.7
NT3	100±7.0	226±7.8	205±19.7	218±19.0
**P14**				
Calb1	100±4.8	96±5.6	101±4.4	48±8.6
Shh	100±6.0	69±5.2	70±4.7	72±5.5
BDNF	100±4.6	78±5.6	59±4.6	97±8.9
NGF	100±8.6	71±1.7	83±5.0	110±4.8
NT3	100±9.6	204±12.1	157±7.6	101±7.1

Data are normalized to the level of rat pups exposed to normoxia at each time point (control 100%). n = 6/group. * $p < 0.05$, ** $p < 0.01$, *** $p < 0.001$, **** $p < 0.0001$ (ANOVA, Bonferroni's post hoc test; Kruskal–Wallis, Dunn's post hoc test; Brown–Forsythe, Dunnett's post hoc test).
