# Supplementary material for: Protective Effect of Dexmedetomidine against Hyperoxia-Damaged Cerebellar Neurodevelopment in the Juvenile Rat
Source: Antioxidants (Basel). 2023 Apr 21;12(4):980. doi: 10.3390/antiox12040980 (PMC10136028; doi:10.3390/antiox12040980)
Supplement: Supplementary file 1 [file antioxidants-12-00980-s001.zip › Table S4. Quantification of cerebellar homogenates for proliferating and migrating GPC factors.pdf]

Table S4. Quantification of cerebellar homogenates for proliferating and migrating GPC–associated mediators.

	NO	NOD	HY	HYD
**hyperoxia**	–	–	+	+
**dexmedetomidine**	–	+	–	+
**P7**				
CycD2	100±4.8	71±8.6	61±7.4	154±9.0
Pax6	100±8.5	53±10.7	52±8.5	161±12.7
Lma1α	100±7.1	36±5.9	45±11.7	47±8.3
Sema6a	100±5.4	63±3.9	69±9.7	96±8.3
**P9**				
CycD2	100±7.4	48±10.4	47±7.0	71±6.5
Pax6	100±5.7	76±13.4	64±6.4	82±15.5
Lma1α	100±6.7	96±8.6	54±8.1	67±7.7
Sema6a	100±5.7	96±6.5	84±1.2	84±8.0
**P11**				
CycD2	100±9.2	125±9.1	161±12.9	97±6.9
Pax6	100±7.2	159±8.6	97±7.6	123±11.3
Lma1α	100±5.9	91±6.5	99±6.9	100±9.8
Sema6a	100±5.8	127±6.1	151±6.8	134±15.3
**P14**				
CycD2	100±5.5	161±15.0	140±18.6	65±12.2
Pax6	100±4.6	154±11.1	129±5.4	42±10.6
Lma1α	100±6.4	80±3.1	81±3.3	134±6.4
Sema6a	100±5.9	85±5.1	79±7.8	56±6.1

Data are normalized to the level of rat pups exposed to normoxia at each time point (control 100%). n = 6/group. * $p < 0.05$, ** $p < 0.01$, *** $p < 0.001$, **** $p < 0.0001$ (ANOVA, Bonferroni's post hoc test; Kruskal–Wallis, Dunn's post hoc test; Brown–Forsythe, Dunnett's post hoc test).
