# Supplementary material for: Protective Effect of Dexmedetomidine against Hyperoxia-Damaged Cerebellar Neurodevelopment in the Juvenile Rat
Source: Antioxidants (Basel). 2023 Apr 21;12(4):980. doi: 10.3390/antiox12040980 (PMC10136028; doi:10.3390/antiox12040980)
Supplement: Supplementary file 1 [file antioxidants-12-00980-s001.zip › Table S5. Quantification of cerebellar homogenates for survival and development GPC factors.pdf]

Table S5. Quantification of cerebellar homogenates for survival and development GPC−associated mediators.

	NO	NOD	HY	HYD
**hyperoxia**	−	−	+	+
**dexmedetomidine**	−	+	−	+
**P7**				
NeuroD1	100±3.6	69±4.9	69±5.3	69±10.1
NeuN	100±3.8	34±2.4	56±8.0	62±5.1
Prox1	100±8.5	97±4.7	74±11.0	89±12.5
Chd7	100±6.0	85±11.1	98±9.1	142±3.6
**P9**				
NeuroD1	100±6.9	56±10.1	57±4.5	83±5.2
NeuN	100±9.1	94±6.8	101±8.0	87±9.5
Prox1	100±10.0	94±10.0	69±5.8	109±7.1
Chd7	100±5.9	79±7.1	94±7.4	72±10.1
**P11**				
NeuroD1	100±8.5	139±8.0	163±12.4	121±5.8
NeuN	100±6.2	130±10.6	142±7.7	186±22.0
Prox1	100±6.4	140±4.4	79±6.2	96±7.5
Chd7	100±9.0	127±7.7	155±9.0	167±16.8
**P14**				
NeuroD1	100±7.0	97±6.7	125±6.2	58±3.9
NeuN	100±6.6	100±4.1	112±1.9	66±8.3
Prox1	100±7.2	146±6.0	82±9.5	52±7.3
Chd7	100±7.5	92±7.2	91±7.4	48±10.9

Data are normalized to the level of rat pups exposed to normoxia at each time point (control 100%). $n = 6$/group. * $p < 0.05$, ** $p < 0.01$, *** $p < 0.001$, **** $p < 0.0001$ (ANOVA, Bonferroni's post hoc test; Kruskal–Wallis, Dunn's post hoc test; Brown–Forsythe, Dunnett's post hoc test).
