# Supplementary material for: Protective Effect of Dexmedetomidine against Hyperoxia-Damaged Cerebellar Neurodevelopment in the Juvenile Rat
Source: Antioxidants (Basel). 2023 Apr 21;12(4):980. doi: 10.3390/antiox12040980 (PMC10136028; doi:10.3390/antiox12040980)
Supplement: Supplementary file 1 [file antioxidants-12-00980-s001.zip › Table S6. Quantification of cerebellar homogenates for survival and development GC factors.pdf]

Table S6. Quantification of cerebellar homogenates for survival and development GC−associated mediators.

	NO	NOD	HY	HYD
**hyperoxia**	−	−	+	+
**dexmedetomidine**	−	+	−	+
**P7**				
Pax2	100±8.9	50±3.1	106±3.8	109±3.4
NeuroD2	100±5.7	61±3.1	60±5.3	132±5.9
Syp	100±10.3	34±7.8	36±5.2	38±3.5
Sox2	100±8.7	34±4.2	49±6.8	50±3.2
**P9**				
Pax2	100±5.1	89±7.3	102±4.7	111±4.9
NeuroD2	100±4.2	65±10.3	69±7.3	73±14.8
Syp	100±5.7	92±5.0	62±4.1	72±6.5
Sox2	100±8.1	113±4.7	77±2.8	84±7.8
**P11**				
Pax2	100±4.0	94±6.4	104±7.5	104±5.3
NeuroD2	100±5.8	117±6.5	126±3.1	87±12.5
Syp	100±2.9	72±3.0	85±1.5	98±5.0
Sox2	100±5.0	95±4.6	108±5.8	116±8.0
**P14**				
Pax2	100±5.9	83±7.1	100±6.7	93±6.7
NeuroD2	100±9.3	94±4.4	115±7.2	59±3.5
Syp	100±6.5	78±3.1	83±4.1	75±2.3
Sox2	100±5.7	87±3.5	100±7.1	106±5.0

Data are normalized to the level of rat pups exposed to normoxia at each time point (control 100%). n = 6/group. * $p < 0.05$, ** $p < 0.01$, *** $p < 0.001$, **** $p < 0.0001$ (ANOVA, Bonferroni's post hoc test; Kruskal–Wallis, Dunn's post hoc test; Brown–Forsythe, Dunnett's post hoc test).
