# Supplementary material for: Protective Effect of Dexmedetomidine against Hyperoxia-Damaged Cerebellar Neurodevelopment in the Juvenile Rat
Source: Antioxidants (Basel). 2023 Apr 21;12(4):980. doi: 10.3390/antiox12040980 (PMC10136028; doi:10.3390/antiox12040980)
Supplement: Supplementary file 1 [file antioxidants-12-00980-s001.zip › Table S7. Quantification of cerebellar homogenates for UBC and CN factors.pdf]

Table S7. Quantification of cerebellar homogenates for UBC− and CN−associated mediators.

	NO	NOD	HY	HYD
**hyperoxia**	−	−	+	+
**dexmedetomidine**	−	+	−	+
		**P7**		
Tbr1	100 ± 7.4	52 ± 7.5	71 ± 1.3	210 ± 35.5
Tbr2	100 ± 3.2	90 ± 5.6	54 ± 3.8	136 ± 6.9
		**P9**		
Tbr1	100 ± 18.0	82 ± 13.1	82 ± 7.6	146 ± 16.6
Tbr2	100 ± 5.5	71 ± 12.0	72 ± 4.7	157 ± 17.7
		**P11**		
Tbr1	100 ± 8.7	163 ± 10.2	77 ± 2.5	120 ± 3.5
Tbr2	100 ± 7.5	161 ± 8.3	89 ± 8.2	150 ± 9.0
		**P14**		
Tbr1	100 ± 6.1	141 ± 14.3	41 ± 6.6	81 ± 7.4
Tbr2	100 ± 6.4	198 ± 10.4	125 ± 4.3	137 ± 8.8

Data are normalized to the level of rat pups exposed to normoxia at each time point (control 100%). n = 6/group. * $p < 0.05$, ** $p < 0.01$, *** $p < 0.001$, **** $p < 0.0001$ (ANOVA, Bonferroni's post hoc test; Kruskal–Wallis, Dunn's post hoc test; Brown–Forsythe, Dunnett's post hoc test).
